# The Brain as a Crystal Ball: The Predictive Potential of Default Mode Network

**DOI:** 10.3389/fnhum.2012.00261

**Published:** 2012-09-26

**Authors:** Stefano Sandrone

**Affiliations:** ^1^Vita-Salute San Raffaele UniversityMilan, Italy; ^2^Neuroimmunology Unit, Division of Neuroscience, Institute of Experimental Neurology, San Raffaele HospitalMilan, Italy

## Introduction

The Danish Nobel Prize Niels Bohr once stated that “making a prediction is very difficult, especially about the future” (Ellis, 1970). The exact and original source of this quotation is still a matter of debate: someone argues that it is an old Danish proverb that Bohr was fond of quoting, others think that this is probably a sentence pronounced by a member of the Danish parliament or perhaps first said by the Danish cartoonist Robert Storm Petersen. Something is rotten in this Danish quote; however, it could be reasonable to agree with the content of the quote itself. Making predictions, one of the basic desires of mankind, is very difficult. Predictions are pillars of scientific method, and, from an epistemological point of view, the holy grail of every scientists should be designing a theory capable of generate predictions on all the possible consequences deriving from the theory itself, with each of them being testable and potentially falsifiable (Popper, 1959). More realistically, some researchers succeed in making, at least, some working hypotheses.

However, something is changing. We currently know that when an individual is awake and alert and not actively engaged in attention-demanding tasks, organized neural activity is present in a set of brain regions called default mode network (DMN; Raichle et al., 2001). This network involves densely interconnected pivotal structures, such as the posterior cingulate cortex (PCC), the precuneus, and parts of the ventromedial prefrontal cortex (vmPFC), and its activity is suspended during specific goal-directed behaviors (Sandrone and Bacigaluppi, 2012). DMN activity have been investigated across the whole range of life cycle, from the emergence in 2-day-old newborn (Gao et al., 2009) to its disappearance in dead brain patients (Boly et al., 2009), through its evolution in both healthy and pathological conditions, where DMN show inter-individual differences, abnormalities, and disruptions (for a review see Power et al., 2010). Interestingly, recent evidence on these DMN differences, abnormalities, and disruptions in both connectivity and activity coming from different neuroscientific domains can account for an emerging concept that we named the “predictive value” or “predictive potential” of DMN (Sandrone and Bacigaluppi, 2012). In fact, it is seems that DMN can be used as predictive behavioral markers and as clinical diagnostic tools, thus making the brain a sort of crystal ball. These predictions are highly heterogeneous and the current evidence are scattered between several neuroscientific domains: they range from neurological to psychiatric conditions, and mix up behavioral phenotypes and neuropsychological evidence, in an intriguing parallelism between connectivity and activity.

## Connectivity within DMN

The connectivity within the posteromedial parts of the precuneus before the execution of a n-back task with increasing levels of memory load, for example, predict working memory execution (Sala-Llonch et al., 2012). DMN predictions can also be projected along the life span and thus be linked to the development trajectory. In juvenile offenders, for example, premotor functional connectivity predicts impulsivity (Shannon et al., 2011). Functional connectivity within the DMN can predict sustained attention ability in Traumatic Brain Injury (TBI) patients (Bonnelle et al., 2011; Sandrone and Bacigaluppi, 2012), and the severity of brain damage induced by mild TBI in the subacute phase of injury can be assessed by alterations in the DMN (Johnson et al., 2012). Moreover, resting state connectivity of the PCC with the perigenual anterior cingulate and the right amygdala in acutely traumatized subjects is associated with PTSD symptoms: the correlation with the right amygdala predicts future PTSD symptoms, possibly setting the stage for DMN based prognostic tools distinguishing between subjects who will and those who will not develop PTSD (Lanius et al., 2010). DMN may also improve early discrimination of dementia with Lewy bodies from Alzheimer disease in cognitively normal individuals (Galvin et al., 2011), and DMN functional connectivity tracks clinical deterioration in Alzheimer patients (Damoiseaux et al., 2012). fMRI connectivity has great utility in predicting future cognitive decline in AD vs MCI, since it indices distinguish patients with MCI who undergo cognitive decline and conversion to AD from those who remain stable over a 2- to 3-year follow up period (Petrella et al., 2011). Increased functional connectivity indicates the severity of cognitive impairment in multiple sclerosis (Hawellek et al., 2011), and may also constitute a robust diagnostic or prognostic metric in individual patients with autism (Anderson et al., 2011). DMN connectivity disruptions in comatose patients may serve as an indicator of the extent of cortical disruption, predict reversible impairments in consciousness (Norton et al., 2012), and reflects the level of consciousness in non-communicative brain-damaged patients. (Vanhaudenhuyse et al., 2010).

## Neural Activity Of DMN

Not only the functional connectivity, but also the neural activation of DMN can have a great predictive value. The neural activity of midline brain regions, including bilateral precuneus and posterior cingulate cortices, perigenual anterior cingulate cortices, and transverse frontopolar gyri, precedes errors during the stop signal task, and, noteworthy, a greater activation of these cerebral areas predicts stop signal errors (Li et al., 2007). In the same psychological test, regional homogeneity of some regions of DMN can predict stop signal reaction time, thus linking once more spontaneous brain activity during resting state to a cognitive performance (Tian et al., 2012). The patterns of longitudinal deficits of DMN may assist investigators to identify and monitor the development of amnestic type mild cognitive impairment (aMCI; Bai et al., 2011). PCC/precuneus dysfunction, in fact, is positively related to the impairments of episodic memory from baseline to follow up in aMCI patients, and thus this DMN hub can be proposed as specifically progressive deficits of brain functional marker (Bai et al., 2011). Default mode network activity distinguishes Alzheimer’s disease from healthy aging, and thus may prove a sensitive and specific biomarker for incipient AD, since Alzheimer disease patients group showed decreased resting state activity in the posterior cingulate (Greicius et al., 2004). Dysfunctions of cerebral networks precede recognition memory deficits in early Parkinson’s disease (PD). PD patients, in fact, showed decreased task-related activations in areas involved in the recognition memory network and decreased task-related deactivations in the DMN in comparison with controls (Ibarretxe-Bilbao et al., 2011). Moreover, Behavioral scores assessing the level of consciousness correlate with deactivation in minimally conscious state and unresponsive wakefulness syndrome patients: deactivation of medial regions can be associated with the level of consciousness, and it may function as a marker of consciousness (Crone et al., 2011).

## Conclusions and Outlines

We are still far from an ultimate understanding of DMN physiology and their functional relevance: the aforementioned “predictive” evidence on both the functional connectivity and neural activity, though pioneeristic, are still puzzling and need to be systematically replicated. Further studies will have mainly to replicate these experiments on larger sample and improve the accuracy rate of every single prediction. However, observing the resting brain to predict a cerebral outcome should be a great challenge for neuroscience (Sandrone and Bacigaluppi, 2012). Discovered in a serendipitous manner (see Buckner, 2012 for a review) because they have been for long time considered a “noise” to be removed and not an informative “signal,” than thought of no utility (Morcom and Fletcher, 2007 but see also Buckner and Vincent, 2007; Raichle and Snyder, 2007), we strongly believe that DMN will be used as predictive tool in several neuroscientific domains. We can not predict whether this cerebral crystal ball will definitively tear down the wall between neurology and psychiatry (Baker et al., 2002) neither we are in front of a paradigm shift (Khun, 1962, but see also Ledford, 2008) that will allow neuroscientists to realize a complete “functional taxonomy” of diseases. Time will tell. Probably Professor Bohr, the popular wisdom, the member of parliament, and the cartoonist are all right: making a (DMN based) prediction is very difficult, but, ultimately, it is not as difficult as it is thought.

## References

[B1] Anderson J. S. Nielsen J. A. Froehlich A. L. Dubray M. B. Druzgal T. J. Cariello A. N. Cooperrider J. R. Zielinski B. A. Ravichandran C. Fletcher P. T. Alexander A. L. Bigler E. D. Lange N. Lainhart J. E. (2011). Functional connectivity magnetic resonance imaging classification of autism. Brain 134, 3739–3751 10.1371/journal.pone.0024271 PMC3235557 22006979

[B2] Bai F. Watson D. R. Shi Y. Wang Y. Yue C. Yuhuan T. Wu D. Yuan Y. Zhang Z. (2011). Specifically progressive deficits of brain functional marker in amnestic type mild cognitive impairment. PLoS ONE 6, e24271 10.1371/journal.pone.0024271 21935394 PMC3174167

[B3] Baker M. G. Kale R. Menken M. (2002). The wall between neurology and psychiatry. BMJ 324, 1468–1469 10.1136/bmj.324.7352.1468 12077018 PMC1123428

[B4] Boly M. Tshibanda L. Vanhaudenhuyse A. Noirhomme Q. Schnakers C. Ledoux D. Boveroux P. Garweg C. Lambermont B. Phillips C. Luxen A. Moonen G. Bassetti C. Maquet P. Laureys S. (2009). Functional connectivity in the default network during resting state is preserved in a vegetative but not in a brain dead patient. Hum. Brain Mapp. 30, 2393–2400 10.1002/hbm.20672 19350563 PMC6870763

[B5] Bonnelle V. Leech R. Kinnunen K. M. Ham T. E. Beckmann C. F. De Boissezon X. Greenwood R. J. Sharp D. J. (2011). Default mode network connectivity predicts sustained attention deficits after traumatic brain injury. J. Neurosci. 31, 13442–13451 10.1523/JNEUROSCI.1163-11.2011 21940437 PMC6623308

[B6] Buckner R. L. (2012). The serendipitous discovery of the brain’s default network. Neuroimage 62, 1137–1145 10.1016/j.neuroimage.2011.10.035 22037421

[B7] Buckner R. L. Vincent J. L. (2007). Unrest at rest: default activity and spontaneous network correlations. Neuroimage 37, 1091–1096; discussion 1097–1099 10.1016/j.neuroimage.2007.01.010 17368915

[B8] Crone J. S. Ladurner G. Höller Y. Golaszewski S. Trinka E. Kronbichler M. (2011). Deactivation of the default mode network as a marker of impaired consciousness: an fMRI study. PLoS ONE 6:e26373 10.1371/journal.pone.0026373 22039473 PMC3198462

[B9] Damoiseaux J. S. Prater K. E. Miller B. L. Greicius M. D. (2012). Functional connectivity tracks clinical deterioration in Alzheimer’s disease. Neurobiol. Aging 33, 828.e19–e30 10.1016/j.neurobiolaging.2011.06.024 21840627 PMC3218226

[B10] Ellis A. K. (1970). Teaching and Learning Elementary Social Studies. Boston: Allyn and Bacon

[B11] Galvin J. E. Price J. L. Yan Z. Morris J. C. Sheline Y. I. (2011). Resting bold fMRI differentiates dementia with Lewy bodies vs Alzheimer disease. Neurology 76, 1797–1803 10.1212/WNL.0b013e31821ccc83 21525427 PMC3100121

[B12] Gao W. Zhu H. Giovanello K. S. Smith J. K. Shen D. Gilmore J. H. Lin W. (2009). Evidence on the emergence of the brain’s default network from 2-week-old to 2-year-old healthy pediatric subjects. Proc. Natl. Acad. Sci. U.S.A. 106, 6790–6795 10.1073/pnas.0811221106 19351894 PMC2672537

[B13] Greicius M. D. Srivastava G. Reiss A. L. Menon V. (2004). Default-mode network activity distinguishes Alzheimer’s disease from healthy aging: evidence from functional MRI. Proc. Natl. Acad. Sci. U.S.A. 101, 4637–4642 10.1073/pnas.0308627101 15070770 PMC384799

[B14] Hawellek D. J. Hipp J. F. Lewis C. M. Corbetta M. Engel A. K. (2011). Increased functional connectivity indicates the severity of cognitive impairment in multiple sclerosis. Proc. Natl. Acad. Sci. U.S.A. 108, 19066–19071 10.1073/pnas.1110024108 22065778 PMC3223469

[B15] Ibarretxe-Bilbao N. Zarei M. Junque C. Marti M. J. Segura B. Vendrell P. Valldeoriola F. Bargallo N. Tolosa E. (2011). Dysfunctions of cerebral networks precede recognition memory deficits in early Parkinson’s disease. Neuroimage 57, 589–597 10.1016/j.neuroimage.2011.04.049 21554963

[B16] Johnson B. Zhang K. Gay M. Horovitz S. Hallett M. Sebastianelli W. Slobounov S. (2012). Alteration of brain default network in subacute phase of injury in concussed individuals: resting-state fMRI study. Neuroimage 59, 511–518 10.1016/j.neuroimage.2011.08.032 21846504 PMC3196274

[B17] Khun T. S. (1962). The Structure of Scientific Revolutions. Chicago: University of Chicago Press

[B18] Lanius R. A. Bluhm R. L. Coupland N. J. Hegadoren K. M. Rowe B. Théberge J. Neufeld R. W. Williamson P. C. Brimson M. (2010). Default mode network connectivity as a predictor of post-traumatic stress disorder symptom severity in acutely traumatized subjects. Acta Psychiatr. Scand. 121, 33–40 10.1111/j.1600-0447.2009.01391.x 19426163

[B19] Ledford H. (2008). Language: disputed definitions. Nature 455, 1023–1028 10.1038/455841a 18948925

[B20] Li C. S. Yan P. Bergquist K. L. Sinha R. (2007). Greater activation of the “default” brain regions predicts stop signal errors. Neuroimage 38, 640–648 10.1016/j.neuroimage.2007.07.021 17884586 PMC2097963

[B21] Morcom A. M. Fletcher P. C. (2007). Does the brain have a baseline? Why we should be resisting a rest. Neuroimage 37, 1073–1082 10.1016/j.neuroimage.2006.09.013 17681817

[B22] Norton L. Hutchison R. M. Young G. B. Lee D. H. Sharpe M. D. Mirsattari S. M. (2012). Disruptions of functional connectivity in the default mode network of comatose patients. Neurology 78, 175–181 10.1212/WNL.0b013e31823fcd61 22218274

[B23] Petrella J. R. Sheldon F. C. Prince S. E. Calhoun V. D. Doraiswamy P. M. (2011). Default mode network connectivity in stable vs progressive mild cognitive impairment. Neurology 76, 511–517 10.1212/WNL.0b013e31820af94e 21228297 PMC3053179

[B24] Popper K. (1959). The Logic of Scientific Discovery. New York: Basic Books

[B25] Power J. D. Fair D. A. Schlaggar B. L. Petersen S. E. (2010). The development of human functional brain networks. Neuron 67, 735–748 10.1016/j.neuron.2010.08.017 20826306 PMC2941973

[B26] Raichle M. E. MacLeod A. M. Snyder A. Z. Powers W. J. Gusnard D. A. Shulman G. L. (2001). A default mode of brain function. Proc. Natl. Acad. Sci. U.S.A. 98, 676–682 10.1073/pnas.98.2.676 11209064 PMC14647

[B27] Raichle M. E. Snyder A. Z. (2007). A default mode of brain function: a brief history of an evolving idea. Neuroimage 37, 1083–1090; discussion 1097–1099 10.1016/j.neuroimage.2007.02.041 17719799

[B28] Sala-Llonch R. Peña-Gómez C. Arenaza-Urquijo E. M. Vidal-Piñeiro D. Bargalló N. Junqué C. Bartrés-Faz D. (2012). Brain connectivity during resting state and subsequent working memory task predicts behavioural performance. Cortex 48, 1187–1196 10.1016/j.cortex.2011.07.006 21872853

[B29] Sandrone S. Bacigaluppi M. (2012). Learning from default mode network: the predictive value of resting state in traumatic brain injury. J. Neurosci. 32, 1915–1917 10.1523/JNEUROSCI.5637-11.2012 22323703 PMC6621708

[B30] Shannon B. J. Raichle M. E. Snyder A. Z. Fair D. A. Mills K. L. Zhang D. Bache K. Calhoun V. D. Nigg J. T. Nagel B. J. Stevens A. A. Kiehl K. A. (2011). Premotor functional connectivity predicts impulsivity in juvenile offenders. Proc. Natl. Acad. Sci. U.S.A. 108, 11241–11245 10.1073/pnas.1108241108 21709236 PMC3131347

[B31] Tian L. Ren J. Zang Y. (2012). Regional homogeneity of resting state fMRI signals predicts stop signal task performance. Neuroimage 60, 539–544 10.1016/j.neuroimage.2011.11.098 22178814

[B32] Vanhaudenhuyse A. Noirhomme Q. Tshibanda L. J. Bruno M. A. Boveroux P. Schnakers C. Soddu A. Perlbarg V. Ledoux D. Brichant J. F. Moonen G. Maquet P. Greicius M. D. Laureys S. Boly M. (2010). Default network connectivity reflects the level of consciousness in non-communicative brain-damaged patients. Brain 133, 161–171 10.1093/brain/awq228 20034928 PMC2801329

